# Neural Representation of Interaural Time Differences in Humans—an Objective Measure that Matches Behavioural Performance

**DOI:** 10.1007/s10162-016-0584-6

**Published:** 2016-09-14

**Authors:** Jaime A. Undurraga, Nick R. Haywood, Torsten Marquardt, David McAlpine

**Affiliations:** 1 Department Linguistics, The Australian Hearing Hub, Macquarie University, 16 University Avenue, Sydney, NSW 2109 Australia; 2 UCL Ear Institute, University College London, 332 Gray’s Inn Rd., London, WC1X8EE UK

**Keywords:** objective measures, behavioural measures, interaural time difference, ethological range, interaural time sensitivity

## Abstract

Humans, and many other species, exploit small differences in the timing of sounds at the two ears (interaural time difference, ITD) to locate their source and to enhance their detection in background noise. Despite their importance in everyday listening tasks, however, the neural representation of ITDs in human listeners remains poorly understood, and few studies have assessed ITD sensitivity to a similar resolution to that reported perceptually. Here, we report an objective measure of ITD sensitivity in electroencephalography (EEG) signals to abrupt modulations in the interaural phase of amplitude-modulated low-frequency tones. Specifically, we measured following responses to amplitude-modulated sinusoidal signals (520-Hz carrier) in which the stimulus phase at each ear was manipulated to produce discrete interaural phase modulations at minima in the modulation cycle—interaural phase modulation following responses (IPM-FRs). The depth of the interaural phase modulation (IPM) was defined by the sign and the magnitude of the interaural phase difference (IPD) transition which was symmetric around zero. Seven IPM depths were assessed over the range of ±22 ° to ±157 °, corresponding to ITDs largely within the range experienced by human listeners under natural listening conditions (120 to 841 μs). The magnitude of the IPM-FR was maximal for IPM depths in the range of ±67.6 ° to ±112.6 ° and correlated well with performance in a behavioural experiment in which listeners were required to discriminate sounds containing IPMs from those with only static IPDs. The IPM-FR provides a sensitive measure of binaural processing in the human brain and has a potential to assess temporal binaural processing.

## Introduction

Binaural hearing confers considerable advantages in everyday listening environments. Comparing the timing and intensity of a sound at each ear allows listeners to locate a sound source on the horizontal plane and to hear out signals in background noise—an important component of ‘cocktail party listening’ (Bronkhorst 2000; Hawley et al. 2004). Sensitivity to interaural time differences (ITDs), in particular, has received considerable attention, due in part to the exquisite temporal performance observed. For sound frequencies lower than about 1.3 kHz, ITDs of just a few tens of microseconds are discriminable at the behavioural level (Garner 1951; Zwislocki and Feldman 1956; Klumpp and Eady 1956a; Brughera et al. 2013). ITDs also contribute to ‘spatial release from masking’—sounds are more easily heard, and speech is more intelligible, when talker and interferer originate from different locations (Licklider 1948); detection thresholds may improve by up to 15 dB for two binaural signals with opposing interaural phase differences (IPDs). Sensitivity to ITDs generally decreases with age (Babkoff et al. 2002) and is typically impaired with hearing loss (Moore et al. 1991), impacting negatively on performance and increasing required listening effort in complex acoustic environments. To this end, measures of binaural function have obvious clinical relevance for the hearing impaired. Given the importance of ITDs to auditory perception, then, and their obvious clinical relevance, there is considerable benefit to be gained from developing objective measures of ITD sensitivity.

However, whilst physiological mechanisms of ITD sensitivity and the limits of neural resolution are widely investigated and increasingly understood, in a range of mammalian (and avian) species (Yin and Chan 1990; McAlpine et al. 2001; McAlpine and Grothe 2003), direct verification of the neural representation of ITDs in the human brain is lacking. Functional imaging studies typically report a complex representation of ITD at the cortical level (Alain et al. 2001; Zatorre et al. 2002; Krumbholz et al. 2005), whilst studies employing electroencephalography (EEG)—potentially useful in clinical settings—have generally assessed ITD sensitivity only tangentially.

The binaural interaction component (BIC)—the difference potential between summed monaural and binaural responses to the same stimuli (Dobie and Berlin 1979)—for example, provides for only an indirect estimate of binaural processing and is largely insensitive to modulations of the ITD within the physiological range (Brantberg et al. 1999) experienced by human listeners (±760 μs; e.g. Constan and Hartmann (2003) and Hartmann and Macaulay (2014)). Alternative methods of assessing binaural sensitivity employing abrupt changes in either the ITD or the interaural correlation of an on-going tone or noise stimulus (e.g. McEvoy et al. (1990) and Chait et al. (2005), respectively) evoke neural markers in the P1-N1-P2 complex of the thalamus and cortex (Ross et al. (2007b))—using magnetoencephalography (MEG); Dajani and Picton (2006)) but assess ITD sensitivity only obliquely, either because of the stimulus type (correlated vs. uncorrelated noise) or the extent of lateralization used to evoke the response (ITDs well beyond the human ethological range, 760 μs).

**FIG. 1:**
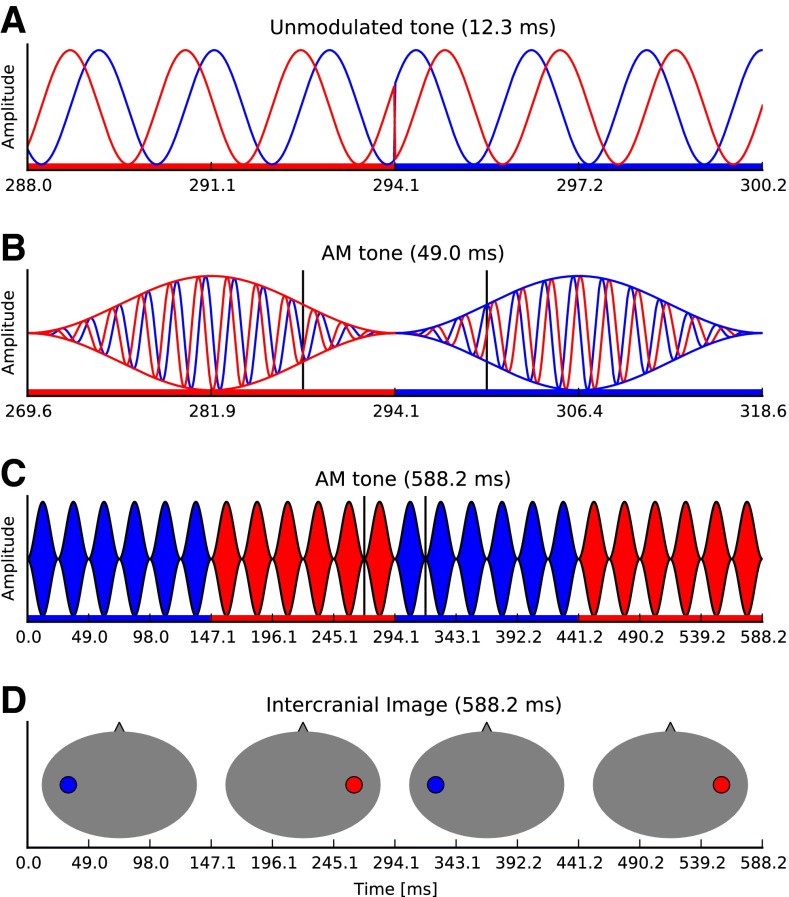
**A** The stimulus before AM. The *blue* and *red lines* correspond to stimuli presented to the left and right ear, respectively. *Filled horizontal bars* indicate the stimulus IPD, with the colour indicating the leading ear. A ±90 ° IPM depth can be observed at 294.1 ms. The *red region* illustrates an IPD of 90 ° (−45 ° to 45 °), whereas the *blue region* illustrates an IPD of −90 ° (45 ° to −45 °). **B** The subsequently introduced AM puts the IPM where the stimulus amplitude is zero in order to minimize monaural cues. The region within the *vertical lines* indicates the time window showed in **A**. **C** Steady IPM. Note that the IPM period contains an integer number of amplitude modulation cycles during which the IPD is held constant. The region within the *vertical lines* indicates the time window showed in **B**. **D** Illustration of perceived intracranial position as a function of the leading phase.

We recently demonstrated that periodic modulations in the IPD of a low-frequency, amplitude modulated (AM) tone (see Fig. 1) where most IPDs were restricted to the physiological range can evoke a steady-state response in human listeners (Haywood et al. 2015; McAlpine et al. 2016). The resulting interaural phase modulation (IPM)—perceived as a sound alternating periodically between left and right intracranial space—evokes a following response (FR) in the EEG signal, which we term the interaural phase modulation following response (IPM-FR). Here, we demonstrate that the magnitude of the IPM-FR varies as a function of the IPM depth and corresponds well with performance of the same listeners in a behavioural task. The data reflect the underlying neural representation of ITD in the human brain with considerably greater resolution than has been demonstrated previously and suggest a potentially robust clinical means of assessing ITD processing in listeners with impaired hearing, or those using hearing devices such as hearing aids and bilateral cochlear implantees.

## Materials and Methods

### Subjects

The initial subject pool consisted of 15 normal hearing (NH) listeners (eight female, mean age = 28, range = 20–47). Two of these subjects were excluded from the analysis as no EEG responses (neither auditory steady state responses (ASSRs) nor IPM-FRs) could be obtained.^1^ None of the listeners reported any known hearing difficulties, and all demonstrated hearing thresholds of 20-dB hearing level (HL) or better for pure tones between 250 and 8000 Hz. The experiment was approved by the University College London Ethics Committee. All subjects provided their informed consent before beginning the experiments and were paid an honorarium for their time.

### Stimuli

The stimuli paradigm was adapted from those employed by Ross and colleagues (Ross et al. 2007a, b; Ross 2008). For all EEG and psychophysical experiments, stimuli comprised a 520-Hz carrier tone sinusoidally amplitude-modulated at 100 %. The AM rate was set to 40.8 Hz, and the amplitude was 65 dB sound pressure level (SPL). Carrier and modulation frequencies were fixed such that an integer number of cycles fitted into an epoch window. Only the carrier was given an IPD, whilst the modulation envelope remained diotic at all times. The magnitude of the IPD was held constant throughout the stimulus, but the ear in which the signal was leading in IPD was periodically alternated between right and left (such modulations, either left-to-right leading or right-to-left leading, are referred to as IPMs). In order to minimize the (monaural) salience of such instantaneous phase transition, IPMs were applied at minima in the AM cycle (see Fig. 1). IPMs were applied periodically at a rate of 6.8 Hz, corresponding to a change in carrier IPD every six AM cycles. This value was chosen as it showed the largest response for most subjects in our initial report and pilot experiments (McAlpine et al. 2016) as well as anticipated to evoke a steady-state response (Dajani and Picton 2006), as opposed to the transient P1-N1-P2 type response as observed by Ross et al. (2007a, b) where responses were evoked by slow (2 s) IPD transitions. This approach has the advantage of requiring a shorter recording session to generate a significant response. Additionally, the response is easy to detect, as the analysis is based on the frequency of interest in the frequency domain, rather than on peak detection in the time domain.

The IPM was symmetrical around zero IPD, i.e. the magnitude of the IPD was the same irrespective of leading ear. For example, an IPM depth of ±45.0 ° refers to the condition in which the IPD was modulated between 45.0 ° and −45.0 °, and for which the overall change in IPD at each IPM was 90 °. In such a case, the IPM depth of ±45.0 ° was generated by advancing the carrier phase at one ear to 22.5 ° and the carrier phase at other ear to −22.5 °. Reversing the sign of the phase at each ear generated the switch in the stimulus IPD between leading at the left and leading at the right ear.

The rate at which these transitions occurred was too high to be lateralized at either side, but it was reported as perceptually salient by all subjects. In total, seven different IPM depths were tested: ±22.5 °, ±45.0 °, ±67.5 °, ±90.0 °, ±112.5 °, ±135.0 ° and ±157.5 ° (corresponding to ITDs of ±120, ±240, ±361, ±481, ±601, ±721 and ±841 μs, respectively).

As stated previously, IPMs were applied at minima in the AM cycle to reduce the salience of changes in the monaural phase. However, in order to verify that EEG-recorded evoked responses were not elicited by such cues, we tested an additional diotic control condition. Here, a diotic stimulus was presented such that no IPD was present, but a uniform monaural phase modulation was applied in both ears, i.e. phase shifts of equal magnitude and direction were applied to both ears, maintaining the IPD at 0 °. The size of the phase change in this control diotic condition corresponded to that in the ±90 ° IPM depth—a condition for which pilot data indicated a large IPM-FR.^2^ Note that stimuli were adapted to measure responses to a range of different IPD values, with the aim of demonstrating that the IPM-FR amplitude varied meaningfully with the magnitude of the IPD, including for IPDs corresponding to ITDs within and outside the human ethological range (±760 μs). IPMs above ±90 ° provide the additional benefit of determining basic mechanisms associated with binaural hearing. Perceptually, IPMs generate a clear intracranial image that switches between left and right sides for IPM depths equal or lower than ±90.0 °. Several studies have demonstrated a linear relationship between IPD and intracranial image location, independent of the frequency (<1200 Hz) (e.g. Sayers 1964; Elpern and Naunton 1964; Yost 1981). However, this relationship does not hold for IPDs beyond 90 ° where the intracranial image either moves towards the midline, jumps to the opposite side, or becomes perceptually diffuse, despite the increasing interaural delay (e.g. Sayers 1964; Elpern and Naunton 1964; Domnitz and Colburn 1977; Shackleton et al. 1991). Thus, one would expect both EEG and behavioural responses to demonstrate a link between behavioural and neural mechanisms of ITD processing.

All stimuli were generated with a custom interface in MATLAB and presented by an RME Fireface UC sound card (24 bits, 48-kHz sampling rate) connected to Etymotic Research ER-2 insert earphones. Sound level was verified with a 2-cm^3^ B&K artificial ear.

### EEG Recordings

Responses were recorded from 66 surface electrodes using a BioSemi Active Two EEG recording system. Sixty-four electrodes were placed in accordance with the international 10–20 system. Two additional electrodes were placed on the left and right mastoid (TP9 and TP10). Electrode voltage offset was typically kept below 20 mV and never exceeded 40 mV.

Responses were recorded at a sampling rate of 16,384 Hz at a resolution of 24 bits/sample (31 nV LSB). The cut-off frequency of the internal low-pass filter was 3334 Hz (5th order sinc response). Recordings were referenced to the vertex electrode and were processed off-line using a custom analysis module in Python 2.7. For the EEG experiments, each of the eight conditions (seven IPM depths, and the diotic control condition) was presented continuously for a total of 5 min and 8 s (75 epochs of 4.109 s). The presentation order of conditions was randomized for each subject. During the recording session, subjects sat in a comfortable chair in an acoustically isolated sound booth and watched a subtitled film of their choice. Subjects were encouraged to sit as still as possible. The total recording time lasted around 40–50 min.

### Data Analyses

#### EEG Processing 

Poor electrode contacts (typically 1 or 2 electrodes containing consistently large or constant amplitudes) were automatically detected and removed from the analysis. After, EEG responses were de-noised using spatial filtering (de Cheveigné and Simon 2008) by the following steps:

1. Epochs from each EEG channel were normalized and submitted to a principal component analysis (PCA), where components with negligible power were discarded. The remaining components were normalized to obtain a set of orthonormal vectors.
2. Epochs were submitted to a bias function. The definition of the bias function determined the rotation matrix obtained on a second PCA, and so its definition depends on the particular problem. Here, we were interested in the response evoked by IPM transitions. Therefore, the bias function was defined as the mean of the frequency component corresponding to the IPM rate (6.8 Hz).
3. A second PCA was applied to data resulting from the bias function. This resulted in a rotation matrix biased towards the evoked response instead of unrelated events such as eye blinks, heart activity, and other on-going brain activity.
4. The rotation matrix resulting from step 3 was applied to the rotation matrix obtained in step 1. The resulting components were ordered by decreasing bias score so that they could be divided into signal components, which were kept, and noise components, which were discarded.
5. Finally, signal components obtained in step 4 were projected back into the sensor space to produce de-noised epochs.

**FIG. 2:**
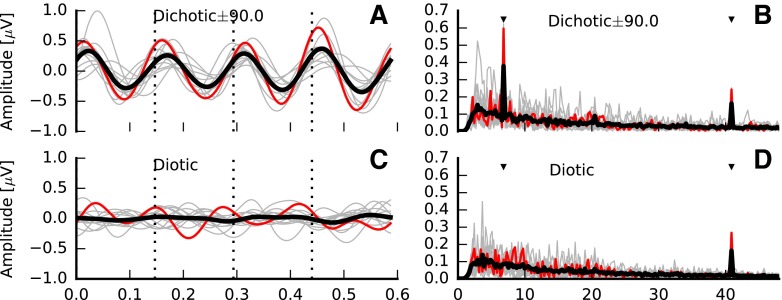
Examples interaural phase modulation following responses (IPM-FRs) for the ±90 ° interaural phase modulation (IPM) depth in the time (**A**) and frequency (**B**) domains for dichotic stimuli. Averaged responses are *highlighted with the thick black lines*, whereas individual responses are shown in *grey*. *Red lines* highlight responses for a single subject (S4). Responses to diotic stimuli are shown on bottom panels **C** and **D**, respectively. All responses correspond to the electrode with the highest signal-to-noise ratio (SNR) for the ±90.0 ° IPM depth. Time domain responses were filtered between 3 and 10 Hz. *Dashed vertical lines* indicate the time at which the IPM occurred. *Black markers* in the frequency domain indicate the IPM rate and the amplitude modulation (AM) rate.

After spatial filtering, epochs of each measurement were transformed to the frequency domain (fast Fourier transform (FFT) of 67,326 points at 0.24-Hz resolution). Two frequency bins were tracked from each epoch, one corresponding to the IPM rate (6.8 Hz) and one to the frequency bin of the AM rate (40.8 Hz; see Fig. 2B, D). The significance of the mean of these frequency bins was evaluated using a two-dimensional repeated measurement Hotelling’s T-squared test (here, confidence intervals are determined from the two-dimensional distribution of the tracked frequency bins, see Picton et al. (1987b) and Picton et al. (2003)) and compared against a significance level of 0.05. In order to obtain clear waveforms in the time domain, IPM-FRs were re-assessed after band-pass filtering the response in the range 3 to 10 Hz so that the presence of the ASSR is minimized whilst facilitating the visualization of the IPM-FR. Per-channel time averages were obtained by applying a weighted averaging method (Don and Elberling 1994). This method estimates the variance of the noise by tracking one or several fixed points over time from a given subset of consecutive epochs. In this study, the power of the residual noise was estimated by tracking 256 equally distant points (1.6 ms), and subsets consisted of a minimum of five epochs. However, the final size was determined adaptively by comparing the variance of successive subsets (Silva 2009). As the variance of each subset is known, the final average is obtained by weighting each subset by the inverse of its variance. This enables all epochs to be used whilst minimizing the effect of non-stationary noise such as eye-blinks artefacts.

### Response Latency Estimation

IPM-FR and ASSR latency responses were estimated following a series of adjustments applied to the phase (*φ*) of the frequency of interest (*f*_*i*_). First, the phase of the frequency bin—*φ*—was adjusted by π rad to set the reference electrode as positive (originally, recordings were referenced to Cz by subtracting this channel). Second, the acoustic delay of 1 ms introduced by insert earphones was computed by multiplying by 2π rad and then dividing by the period of the respective frequency. Third, ASSRs were additionally adjusted by π/2 rad in order to compensate for the frequency transformation, which was computed in terms of a cosine phase, whilst the amplitude was modulated using a sinusoidal function. For IPM-FRs, it was assumed that the abrupt IPM generated a P1-N2-P2 pattern similar to those obtained by abrupt transitions in the interaural correlation of noise (Dajani and Picton 2006) or in IPD (Ross et al. 2007b). As the abrupt transition had zero phase, i.e. the IPM was applied periodically starting at zero time, no adjustment was applied. Once all adjustments were applied, the adjusted phase (*φ*_*a*_) was used to compute the phase delay (*φ*_*d*_) as:

1 $$ {\varphi}_d=2\pi -{\varphi}_a $$

The response latency was estimated as

2 $$ L=\frac{1}{2\pi \times {f}_i}\left({\varphi}_d+2\pi \cdot n+2\pi \cdot m\right) $$

where *n* and *m* are integer numbers. Here, *n* is estimated to account for a circularity ambiguity (phase unwrapping) associated to phase noise. That is, if the neural response is precisely phase-locked a few radians above zero, noise may result in phase fluctuations just below zero radians which, due to circularity, will be estimated a few radians below 2π. On the other hand, *m* is set to account for the number of signal cycles occurring before the response is evoked. In this study, ASSRs were adjusted by setting *m* to 1 as it has been shown that this value corresponds well with phase-delay estimations using several modulations frequencies (for details refer to Rodriguez et al. 1986; John and Picton 2000; Herdman et al. 2002). For IPM-FRs, *m* was 15 set to zero as the response was not expected to occur after 147.0 ms (the period of the IPM rate).

### Psychophysical Experiment

In an adaptive two-interval, two-alternative forced choice task, subjects were required to identify a target tone containing IPMs from a reference tone with an equivalent static IPD. The degree of difficulty with this task was controlled by presentation of pink (−3 dB per octave) masking noise, which was synchronously switched on and off with the reference and target signal. Tone duration was set to 1.024 s. The interval containing the target sequence was chosen randomly on each trial. There was a 1-s pause between the two intervals, and a 1-s pause after subjects responded. Subjects responded via computer keyboard and received visual feedback to indicate correct/incorrect responses. The level of the masking noise was adjusted with a three-up, one-down adaptive staircase procedure so that detectability approached the 79.4 % correct point on the psychometric function (Levitt 1971). The masking noise was low-pass filtered (2 kHz) and interaurally uncorrelated. The level of the modulated tone was fixed at 65 dB SPL. The initial step size was 3 dB, and after two reversals, the step size was reduced to 1 dB and then held constant for four further reversals. Each run comprised six reversals in total. Runs would terminate prematurely if the listener made six incorrect responses at the starting noise level, and this maximal value was taken as the estimate of threshold. In total, 15 out of 315 runs (15 listeners × 7 IPM depth × 3 repetitions) were terminated in this manner. The mean level of the final four reversals was taken as the masking threshold for that run. Prior to the main experiment, subjects completed a brief training session that comprised four of the experimental conditions across the range of IPM depth tested (i.e. ±22.5 °, ±67.5 °, ±112.5 ° and ±157.5 °). Subjects were not screened on the basis of training performance, but the starting level of the noise was set to 6 dB below the highest threshold observed in the four training conditions. The main experiment was split into blocks. Each block comprised a single run of each condition, and the presentation order of the conditions was randomized for each block. Subjects completed three blocks in total. The threshold value was estimated from the mean threshold across the three repetitions per condition. However, if the standard deviation of the three threshold estimates was greater than 4 dB, the listener completed an extra run at the end of the experiment, and the outlying estimate of the four values was excluded from the dataset. Additionally, if the standard deviation of the final four turn-points within a run was greater than 2 dB, an extra run of that condition was completed at the end of the experiment to replace the initial run. Any additional runs were presented at the end of the main experiment and were presented in a random order. In total, 14 runs were repeated for these reasons.

Each trial block lasted for approximately 30 min, and listeners typically completed the behavioural experiment in two or three separate sessions.

The total duration of the experiment was approximately 2.5–3 h.

### Statistical Analysis

In order to perform analysis of variance (ANOVA) with unbalanced data sets, linear mixed-effects (LME) models were used. LME models can deal with unbalanced data, complex modelling of random effects variables, and can account for non-sphericity (Krueger and Tian 2004; Baayen et al. 2008; Bates et al. 2015). LME models with more than one factor were fitted and then followed by a backward stepwise reduction method in order to remove non-significant factors. The degrees of freedoms of the model were estimated by means of Satterthwaite approximation, and the significance level was set to *α* = 0.05. Conditional, random, and marginal residuals of the LME model were checked by visual inspection. When outliers were detected, these were removed and the LME model was refitted. For all LME models in this study, the factor subject was set as random. All statistical analyses were performed using the R software package (R Development Core Team 2015).

## Results

A total of 13 subjects contributed to the data. Two other subjects were excluded from all analyses, including psychoacoustics, as they failed to show a clear evoked ASSR to the 40.8 Hz AM.

### Sensitivity of the EEG to IPM

We measured IPM-FRs for seven different values of IPM depths (±22.5 °, ±45.0 °, ±67.5 °, ±90.0 °, ±112.5 °, ±135.0 ° and ±157.5 °), where the IPM was centred at 0 ° IPD.

Examples of IPM-FRs are shown in Fig. 2. Figure 2A shows responses in the time domain (the time window was 4 IPMs to facilitate visual inspection), for all 13 listeners individually (thin grey lines) and the average response (thick black line), to a single IPM depth (±90.0 °). In order to facilitate the following up of a single case, responses of a single subject (S4) are highlighted here and throughout the entire manuscript in red. These responses were obtained at the best recording electrode—defined as that showing the highest signal-to-noise ratio (SNR), which varied across listeners. Both the individual responses and the average response clearly follow the 6.8 Hz IPM. Note that for this ±90 ° IPM depth, the equivalent change in ITD is ±481 μs—well within the human ethological range of ±760 μs. Figure 2B shows the frequency domain response for all 13 listeners individually (thin grey lines). The individual case in Fig. 2A is highlighted in red and the group-average response is shown with the thick black line. Two peaks are prominent in the FFT: a peak at 40.8 Hz—the ASSR to the AM rate; and a peak at 6.8 Hz—the IPM-FR. In contrast to the dichotic condition, in which phase transitions in the signals of each ear were of identical magnitude, but opposite sign, no IPM-FR was observed for the diotic control condition, in which the IPD remained zero because the phase transitions were of the same magnitude, but of identical sign in both ears (Fig. 2C, D). Note that a prominent ASSR to the 40.8 Hz AM was still evident in these diotic control responses.

**FIG. 3:**
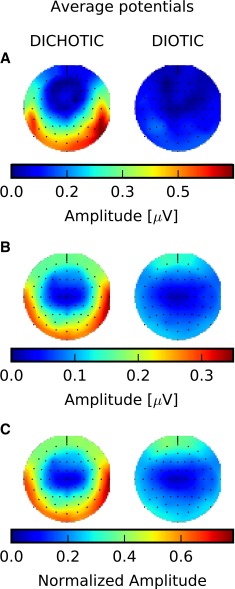
Spectral amplitude across scalp electrodes obtained for the interaural phase modulation following response (IPM-FR) (6.8-Hz frequency bin) for dichotic (±90.0 ° interaural phase modulation (IPM) depth, *left*) and diotic (no IPM, *right*) conditions, respectively. **A** Spectral amplitude for a single subject (S4). **B** Average spectral amplitude across all subjects. **C** Average of the normalized individual spectral amplitudes across all subjects. Responses for each subject were normalized by the maximum spectral amplitude across all conditions from that particular subject so that the normalized average minimizes across-subject variability in the response strength. The anterior (nose) is on the *top*, the posterior (*backside of the head*) is at the *bottom*, and the *left side* corresponds to the left side of the subjects. Spectral amplitudes for areas between adjacent electrodes or areas with discarded electrodes were estimated by mean of cubic interpolation.

The same data are also plotted in Fig. 3 in the form of the spectral amplitude (6.8 Hz frequency bin) across the scalp for the dichotic (left panels) and diotic (right panels) conditions. Once more, scalp spectral amplitudes to the dichotic stimuli are observed for the single participant (Fig. 3A), the group average (Fig. 3B), and the average of the normalized individual responses Fig. 3C (used to minimize the effect of across-subject variability in the overall response strength), whilst the diotic condition did not evoke significant responses at any electrode site (as indicated by the Hotelling’s T2 test). This confirms that the IPM-FR is truly a measure of ITD sensitivity and is not generated by some other factor such as the instantaneous diotic phase switches—which also occur at a rate of 6.8 Hz.

**FIG. 4:**
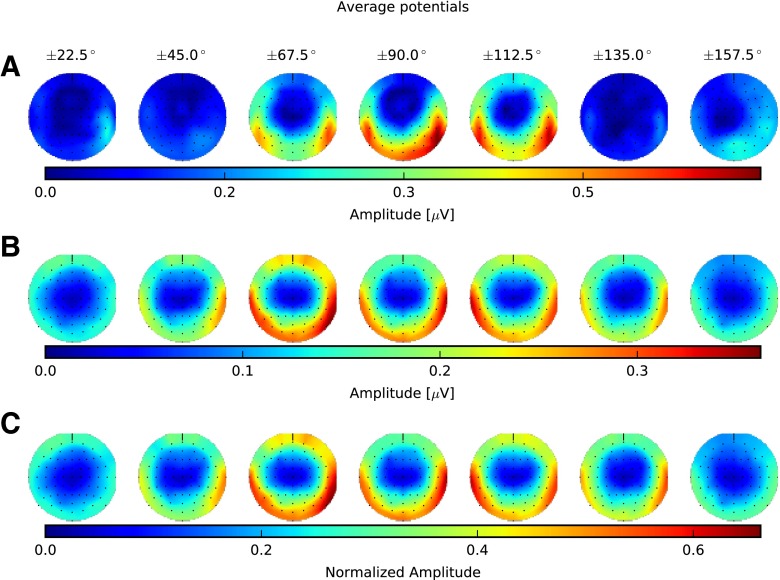
Spectral amplitude across scalp electrodes obtained for the interaural phase modulation following response (IPM-FR) (6.8-Hz frequency bin) for all dichotic interaural phase modulation (IPM) depths. **A** Spectral scalp potentials for a single subject (S4). **B** Average of spectral amplitude across subjects. **C** Average of the normalized individual spectral amplitude across subjects. IPM-FRs for each subject were normalized by the maximum spectral amplitude across all IPM depths from that particular subject so that the normalized average minimizes across-subject variability in the response strength. The corresponding IPM depths are indicated *above the top row*. The anterior (nose) is on the *top*, the posterior (*backside of the head*) is at the *bottom*, and the *left side* corresponds to the left side of the subjects. Spectral amplitudes for areas between adjacent electrodes or areas with discarded electrodes were estimated by mean of cubic interpolation.

In terms of equivalent ITD, the smallest IPM depth we assessed was ±22.5 °, corresponding to ±120 μs, roughly three to four times the ITD discrimination thresholds obtained for an average listener using short (300 ms to 500 ms) tone bursts (e.g. Hershkowitz and Durlach 1969; Klumpp and Eady 1956b; Brughera et al. 2013) and similar to IPD discrimination thresholds in normal listeners using long (1.4 s to 2 s) tone bursts (Hopkins and Moore 2010, 2011; King et al. 2014). In general, we found that the amplitude of the IPM-FR first increased with increasing IPM depth but then declined again. This is evident from Fig. 4 which shows the spectral amplitude of the IPM-FR (6.8 Hz frequency bin) across the scalp for all IPM depths. Both the amplitude and electrode pattern of the response were variable across IPM depth and subjects. IPM-FRs for S4 (Fig. 4A) were greatest in amplitude for the IPM depth of ±90 ° and strongest on posterior and temporal electrodes, whilst the ±112.5 ° IPM depth was strongest on temporal electrodes. Nevertheless, group average (Fig. 4B) and average of the normalized individual responses (Fig. 4C) showed similar tuning to the depth of the IPM, with highest responses for IPM depths in the range of ±45 ° to ±112.5 °.

**FIG. 5:**
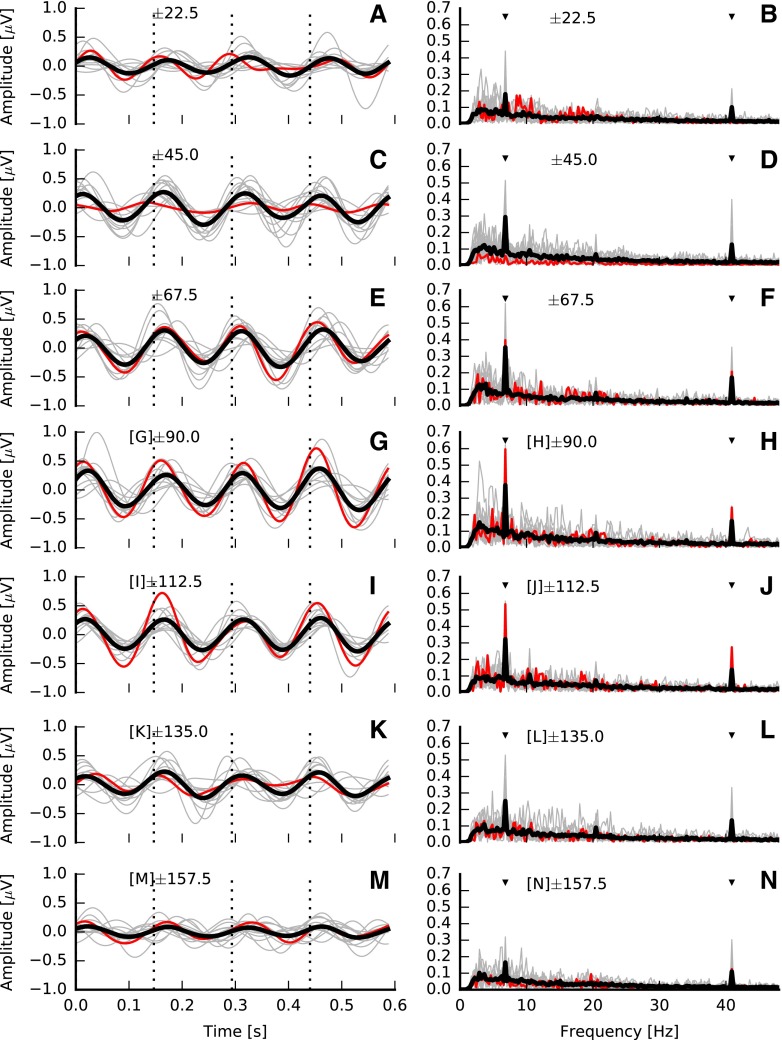
Grand-averaged responses in the time (*left column*) and frequency (*right column*) domains to dichotic stimuli. Grand-averaged responses are highlighted with the *thick black lines*, whereas individual responses are shown in *grey*. *Red lines* highlight responses for subject S4. All responses correspond to the electrode with the highest signal-to-noise ratio for each interaural phase modulation (IPM) depth. Time domain responses were filtered between 3 and 10 Hz. *Dashed vertical lines* indicate the time at which the interaural phase difference transition occurred. *Black markers* in the frequency domain indicate the IPM rate and the amplitude modulation rate.

**FIG. 6:**
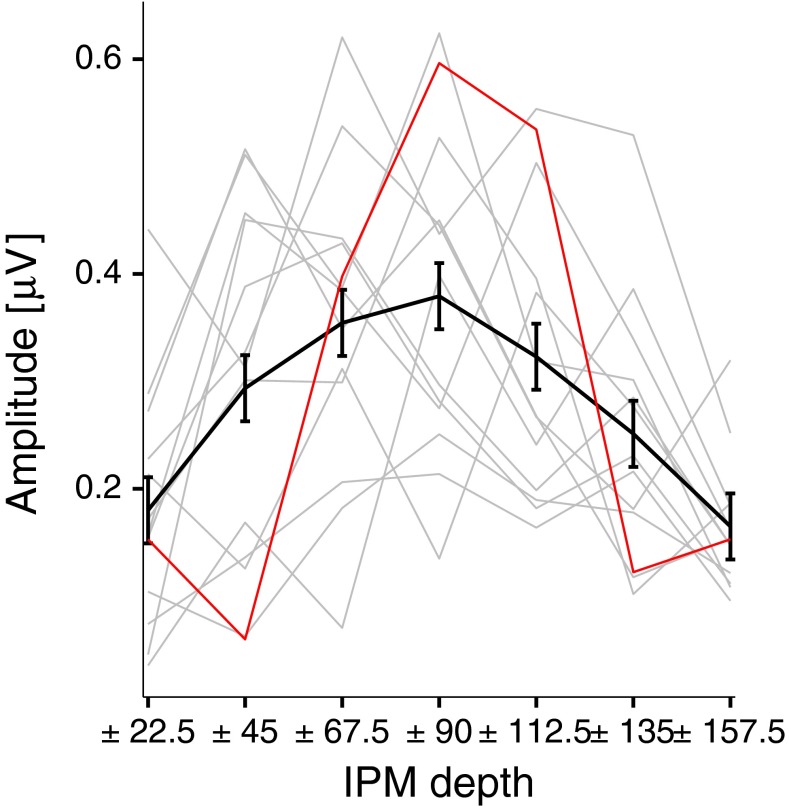
Interaural phase modulation following response (IPM-FR) spectral amplitude as a function of interaural phase modulation (IPM) depths. The *thick black line* corresponds to the grand-averaged response. Individual responses are shown in *grey*. The *red line* highlights the response for subject S4. *Error bars* correspond to Fisher’s least significant difference to facilitate visual post hoc comparisons.

This is confirmed in Fig. 5, which plots responses in the time domain and the spectral amplitude for all IPM-FRs for all conditions. Once again, S4 is highlighted in red and the average is shown with the thick black line. Clear IPM-FRs were obtained for all IPM depths, but with higher amplitude around ±90 ° IPM depth (replotted in Fig. 6 for clarity).

A one-way repeated measures ANOVA applied to the IPM-FRs spectral amplitude with the highest SNRs across all electrodes (Fig. 6) indicated that the IPM depth had a significant effect on the amplitude of the IPM-FR (*F*(6, 72) = 7.1, *p* < 0.001). Note that the smallest IPM-FR elicited was in response to an IPM depth of ±157.5 ° which corresponds to an equivalent ITD excursion of ±841 μs, greater than the human ethological range of ±760 μs for an average listener.

### IPM-FRs Measured across Electrode Locations

As shown in Fig. 4, IPM-FRs were reliably obtained from electrodes located near the temporal, parietal, and occipital lobes, particularly so for IPM depths between ±45.0 ° and ±112.5 °. The analysis indicates that 44 of 82 IPM-FRs had their best SNR at an electrode located on the right hemisphere, whilst only 31 were on the left hemisphere and 7 on the midline. A paired *t* test indicated that this hemisphere difference (left vs. right) was significant (*t* = −3.7, *p* < 0.001).

**FIG. 7:**
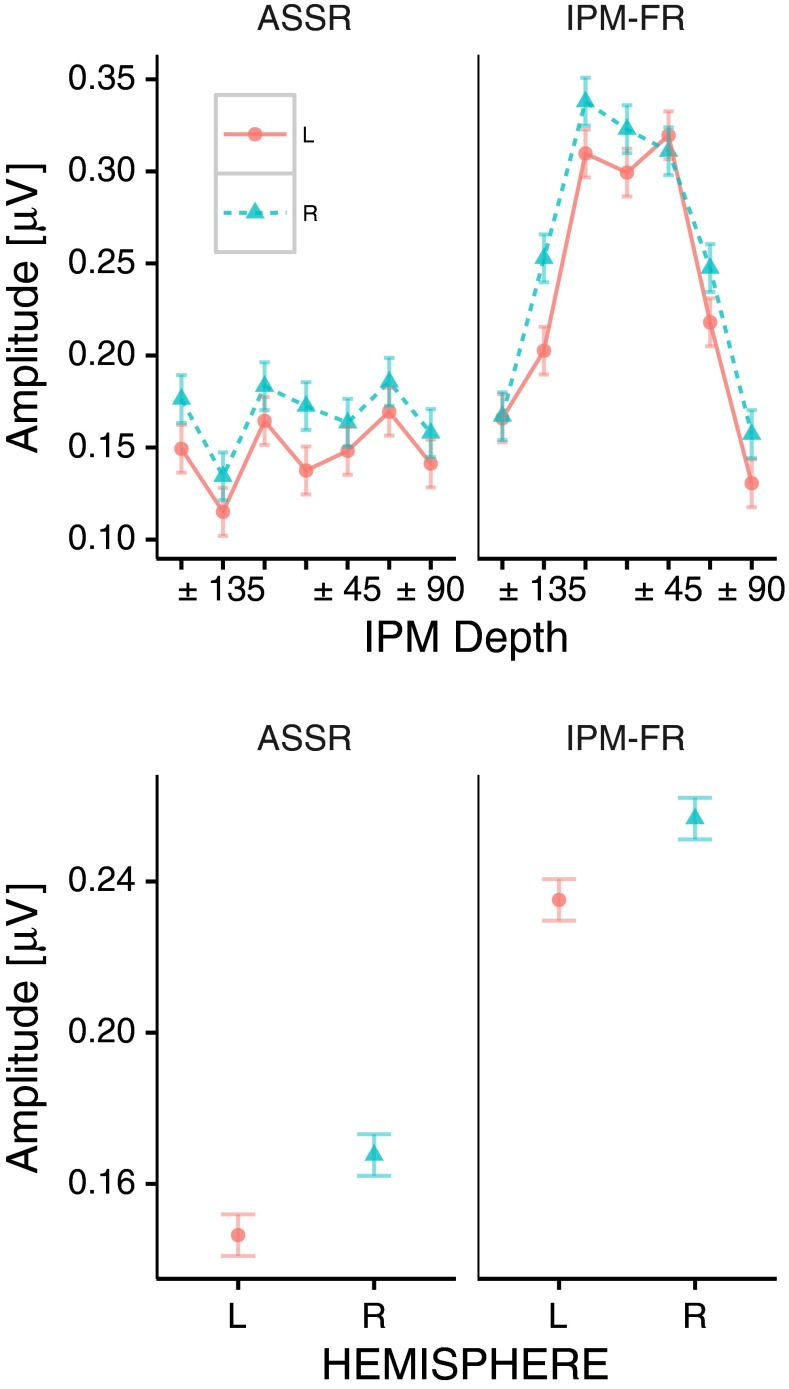
Hemispheric imbalance for interaural phase modulation following responses (IPM-FRs) and auditory steady state responses (ASSRs). Best ASSRs and IPM-FRs for all interaural phase modulation (IPM) depths (*top*). Across IPM depths averages for left (*L*) and right (*R*) hemispheres (*bottom*). Data included the three electrodes with the largest signal-to-noise ratio at each hemisphere. *Error bars* correspond to Fisher’s least significant difference to facilitate visual post hoc comparisons.

In order to test the influence of brain hemisphere on the number of significant recordings, a three-way repeated measures ANOVA (including response type ASSR and IPM-FR, IPM depth, and hemisphere) was applied to the average spectral amplitude of the three best electrodes in either hemisphere (in terms of SNR) at each hemisphere. This analysis indicates that hemisphere (*F*(1, 12) = 14.24, *p* = 0.002), response type (*F*(1, 12) = 15.02, *p* = 0.002), IPM depth (*F*(6, 72) = 6.45, *p* < 0.001), and the interaction between response type and IPM depth (*F*(6, 72) = 11.11, *p* < 0.001) were significant factors (top panel in Fig. 7).

On average, the IPM-FR from the right hemisphere was ≈9 % higher than that obtained from the left hemisphere and ≈ 14 % higher for the ASSR (bottom panel in Fig. 7).

The interaction between response type and IPM depth reveals that, overall, ASSRs are less affected by the IPM depth than IPM-FRs.

### Latency estimations

**FIG. 8:**
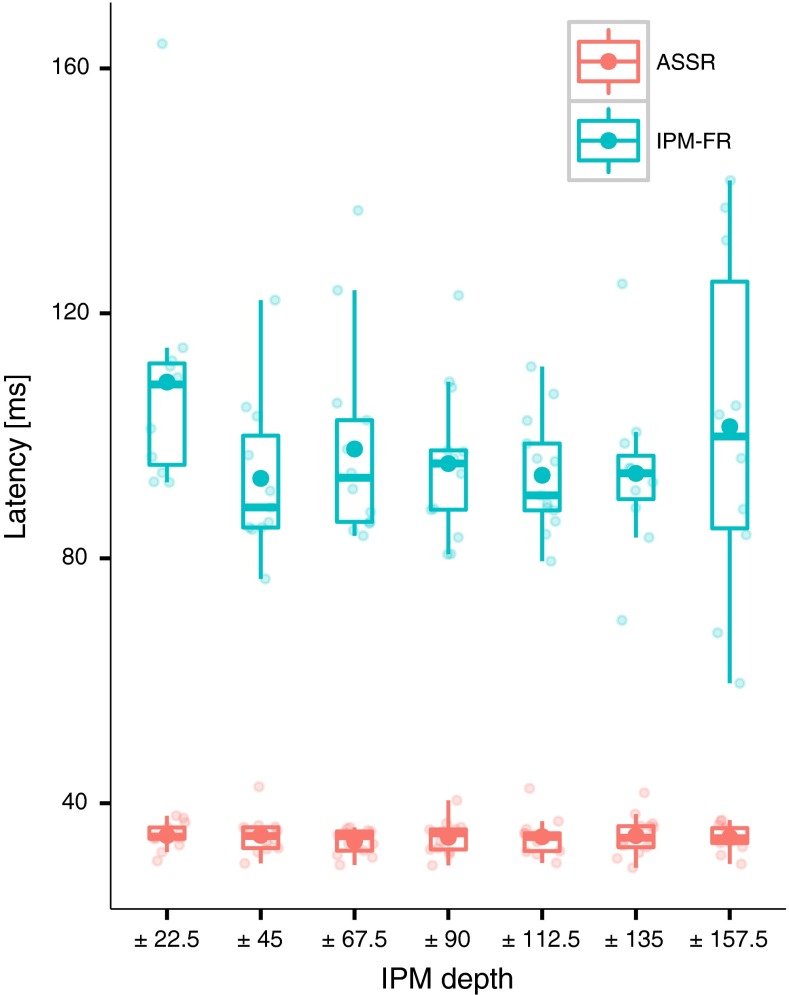
Latency estimations for both interaural phase modulation following responses (IPM-FRs) and auditory steady-state responses (ASSRs). Latencies were estimated by means of Eq. 2. The *small transparent circles* correspond to individual latencies, whilst the *solid filled circles* correspond to the mean latency across subjects. The *upper* and *lower hinges of the box plots* correspond to the first and third quartiles, whilst median is indicated by the *horizontal line within each box plot*.

As described in the methods, the latency of both ASSRs and IPM-FRs was estimated for all significant responses at the electrode location with the highest SNR. These are shown in Fig. 8 and it can be observed that ASSRs were, overall, very similar across subjects and IPM depths (as shown by the box plots). A LME model with IPM depth as a fixed factor did not reveal a significant effect on the ASSR latency (*F*(6, 68.06) = 1.55, *p* = 0.17). The mean latency was 34.48 ms (SD 2.59 ms; range 29.47–42.69 ms) across all IPM depths, and it agrees well with studies using similar modulation frequencies (e.g. Stapells et al. 1984; Picton et al. 1987a).

As observed in Fig. 8, IPM-FR latencies were more variable across IPM depth. The mean latency was 97.52 ms (SD 16.79 ms; range 59.60–164.03 ms) across all IPM depths. A LME model with IPM depth as a fixed factor indicated that the IPM depth had a significant effect on the IPM-FR latency (*F*(6, 54.4) = 4.10, *p* = 0.001). On average, the ±22.5 ° and ±157.5 ° IPM conditions were 10.36 and 5.87 ms larger, respectively, than all other conditions. This would be expected as a consequence of reduced neural synchronization for those conditions producing weak responses.

### Psychophysical Assessment of Sensitivity to IPMs

To compare our objective measure of ITD sensitivity with subjective performance, we assessed the ability of the same subjects as partook in the EEG recordings to discriminate IPM’ed from otherwise identically amplitude-modulated tones containing static IPDs. In an adaptive two-interval, two-alternative forced choice task, subjects were required to identify a target tone containing IPMs from a reference tone with an equivalent static IPD in the presence of a binaurally uncorrelated noise. The dependent variable under investigation was the level of masking noise required to obtain threshold performance (see “Materials and Methods” for further details).

**FIG. 9:**
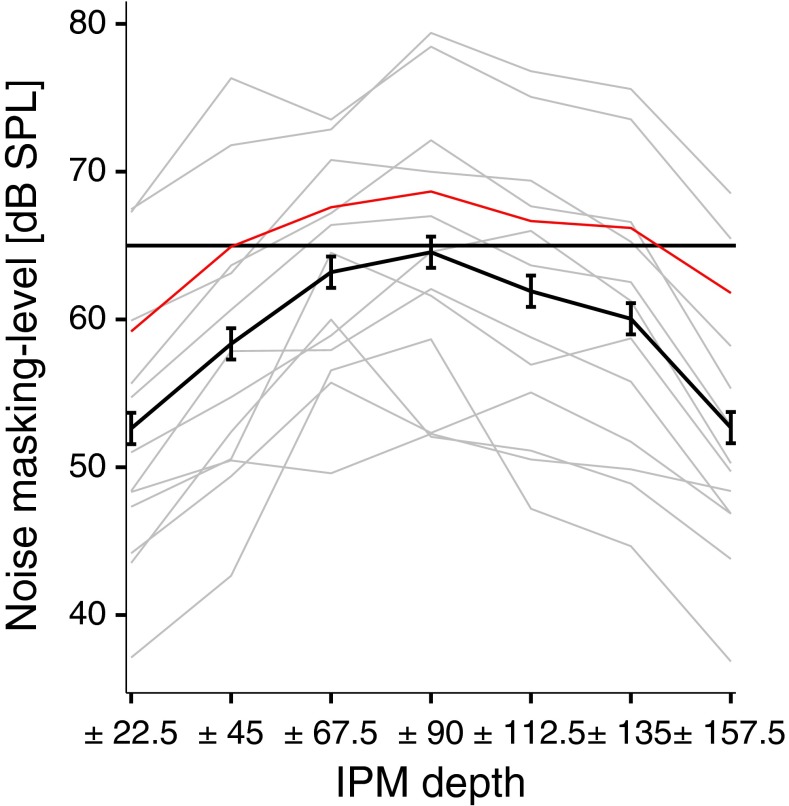
Individuals (*grey lines*) and mean (*black line*) interaural phase modulation (IPM) masking levels for psychophysics data and all IPM depths tested. The *red line* highlights the response for subject S4. The *horizontal line* indicates the level of the modulated tone. *Error bars* correspond to Fisher’s least significant difference to facilitate visual post hoc comparisons.

Figure 9 shows the IPM discrimination thresholds for individual subjects (thin grey and red (S4) lines) and the across-subject average (thick black line), in terms of masking-noise levels in dB for each IPD condition. The maximum masking level across all IPM depths was variable across subjects (≈27.1 dB difference, SD = 8.2 dB). The difference between the maximum (usually for the ±90 ° IPM depth) and the minimum (either the ±22.5 ° or ±157.5 ° IPM depth) masking level was also variable across subjects. The smallest difference was 3.9 dB (S11), and the largest difference was 21.8 dB (S1). Nevertheless, most subjects tolerated higher levels of masking noise for IPM depths between ±67.5 ° and ±112.5 °, and lower levels of masking noise for either ±22.5 ° or ±157.5 °. Across-subject mean data indicate that the ±90.0 ° IPM depth required a higher masking level to obscure the presence of IPM than the other conditions (±90.0 ° masking level = 64.5 dB SPL), which we interpret to indicate that this IPM was the most perceptually salient condition for the majority of subjects (8/13). As the IPM depth departed from ±90.0 °, a progressively lower level of noise was required to mask the presence of the IPM. The two most extreme IPM depths, ±22.5 ° and ±157.5 °, were most susceptible to masking noise with masking levels of 52.0 and 52.2 dB SPL, respectively. Thus, the highest average masking level (observed in the ±90.0 ° IPM depth) was about 12 dB higher than either at ±22.5 ° or ±157.5 °. A one-way repeated-measures ANOVA confirmed a significant main effect of IPM depth (*F*(6, 72) = 41.10, *p* < 0.001).

### Relation between Objective and Behavioural Measures

The average amplitude of the objectively determined IPM-FR and behaviourally determined masking level threshold for IPM discrimination (Figs. 6 and 9) demonstrate a similar pattern of results. IPM-FR amplitudes and masking levels were highest for IPM depths between ±67.5 ° and ±112.5 ° in an inverse U-shaped fashion.

**FIG. 10:**
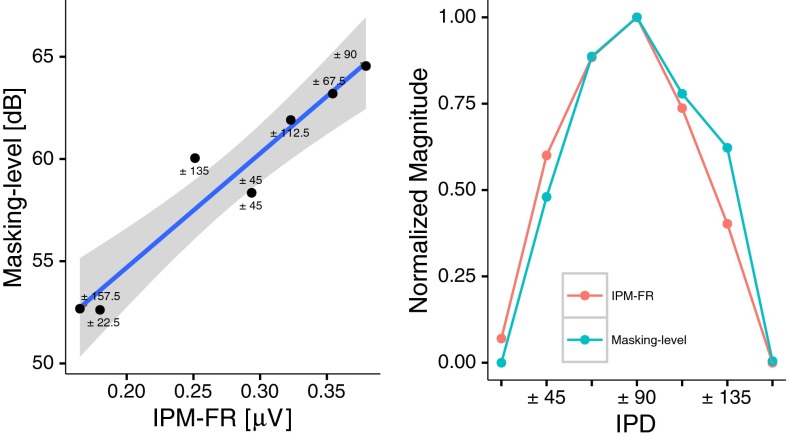
Correlation between mean interaural phase modulation following response (IPM-FR) and behavioural interaural phase modulation (IPM) masking levels (*left*). *Circles* correspond to the mean values for both IPM-FR amplitude (*horizontal axis*) and masking level (*vertical axis*) at a given IPM depth (indicated for each data point). The *grey area* indicates the confident intervals of the regression line, shown in *blue*. The *bottom axis* shows the different interaural phase differences tested, whilst the *vertical axis* shows the normalized, non-dimensional, mean amplitude across subjects for each measurement (*right*).

Across subjects average responses are shown in the right panel of Fig. 10 in terms of a unitary scale to facilitate visual comparisons. Both measures generated very similar functions. Indeed, the statistical analysis indicated that these two measures are strongly correlated (*R* = 0.96, *p* < 0.001, left panel in Fig. 10).

At subject level, preliminary analyses using the original raw data points indicated that only 5 out of 13 subjects demonstrated significant correlations between these two measures. This was expected because of the reduced number of degrees of freedom rendering regression susceptible to small deviations resulting from noise as opposed to the mean data where the noise was reduced by averaging over all subjects. Thus, IPM-FRs and masking level data were smoothed independently by fitting second-order polynomial functions by means of maximum-likelihood estimation. A second-order polynomial fitting was chosen as this function seems to match well the mean data shown in Fig. 10.

**FIG. 11:**
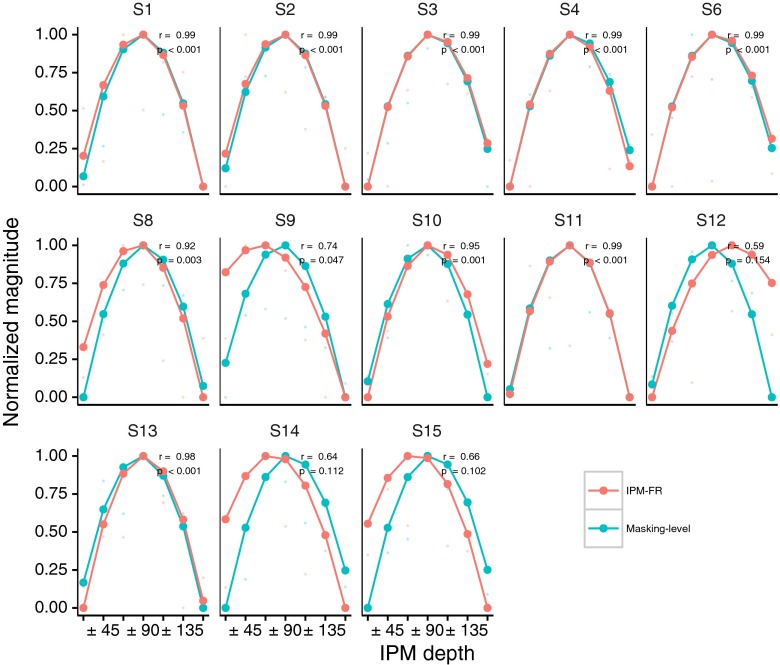
Correlations between interaural phase modulation following response (IPM-FR) and behavioural interaural phase modulation (IPM) masking levels for all subjects. The *bottom axis* shows the different interaural phase differences tested, whilst the *vertical axis* shows the normalized, non-dimensional, amplitude responses of both measurements. *Single data points* correspond to the raw data and *solid lines* correspond to the fitted second order polynomial data. Pearson’s correlation coefficients (*r*) and *p* values (*p*), obtained from the smoothed unnormalized data, are indicated for each subject.

The data for all 13 subjects were normalized as shown in Fig. 11 and indicate that IPM-FR and behavioural noise masking threshold levels generally correlate well with each other.

Pearson’s correlation coefficients (*R*) ranged from 0.59 to 0.99 (mean = 0.88, SD = 0.16) and resulted in significant correlations for 10 of the 13 subjects. Overall, the models’ residuals were well behaved, i.e. normally distributed as confirmed by Shapiro’s test of normality and uncorrelated as confirmed by Durbin-Watson autocorrelation test.

A LME model was fitted in order to investigate whether a relationship existed between the amplitude of the IPM-FR and the masking level at a given IPM depth. The model included the IPM-FR amplitude as the dependent variable, and masking level and IPM depth as fixed factors, and indicated that the IPM depth was the only significant factor (*F*(6, 72) = 7.1, *p* < 0.001). Neither masking level nor the interaction between IPM depth and masking level were significant, suggesting that the masking level and the amplitude of the IPM-FR, at a particular IPM depth, are not related, i.e., large masking levels do not imply large IPM-FRs. This was corroborated by correlation analyses between IPM-FRs and masking levels at each IPM depth. The results indicated that all intercepts (the mean at a given IPM depth) were significant (*p* < 0.001) but none of the slopes were, suggesting that the amplitude of the IPM-FR is not related, at least under the current stimuli parameters, to mean masking level.

In conclusion, these data demonstrate that the objectively measured IPM-FR as a function of the IPM depth reflects the interaural temporal processing capabilities as they are observed behaviourally.

## Discussion

Our data demonstrate a strong agreement between psychoacoustic and objective measures of ITD sensitivity in human listeners. The neural measures correlated well with performance in a binaural listening task in which listeners asked to discriminate stimuli containing IPM from those containing static IPDs of the same magnitude. These results extends our initial reports (Haywood et al. 2015; McAlpine et al. 2016) that IPM-FRs are an easily determined objective measure of sensitivity to ITDs conveyed by low-frequency tones in the EEG response of normal-hearing listeners. The IPM-FR appears to be a direct measure of ITD sensitivity.

The Neural Generators of the IPM-FR

As shown in Fig. 8, IPM-FR had a mean latency of 97.52 ms spanning 59.60 to 164.03 ms. This lies well within the range of latencies of P1-N1 potentials observed in EEG and MEG studies, where the main sources have been identified at the Planum temporale (PT) and the Heschl’s gyrus (HG) (Liégeois-Chauvel et al. 1994; Yvert et al. 2005; Ross et al. 2007b; Yamashiro et al. 2011). Similarly, von Kriegstein et al. (2008)—using functional magnetic resonance imaging (fMRI)—have shown that ITDs of 500 μs produced stronger activation in the hemisphere (PT) contralateral to the lateralized location, whilst ITDs of 1500 μs produced similar activation in both hemispheres (PT and HG). This suggests that both PT and HG are the main sources generating the IPM-FR. If this is indeed the case, the strong correlation between the IPM-FR and the behavioural task suggests that these areas may be involved in the perceptual discrimination of auditory changes.

Although the IPM-FR is likely generated by neurons in cortical brain areas, its sensitivity to binaural cues reflects processing generated in the binaural brainstem nuclei, i.e. the medial superior olive (MSO) and lateral superior olive (LSO), nuclei dedicated to processing binaural time and level differences, respectively. An important consideration in our study concerns the ‘extent of laterality’ of low-frequency tones containing IPDs. When heard over stereo headphones, the extent to which a tone is heard to be lateralized to one side or the other increases with increasing IPD, up to about 90.0 °, before the (intracranial) sound image starts to move back towards the midline (Yost 1981; Shackleton et al. 1991). This has been taken to reflect the underlying neural architecture of ITD processing (Thompson et al. 2006). The close correspondence of preferred IPMs depth in the IPM-FRs and the psychophysical task suggests that both measures reflect this well-established aspect of binaural hearing.

In a previous study using MEG, Ross et al. (2007b) presented a 4-s amplitude-modulated tone in which the IPD was modulated instantaneously from zero to 180 ° (anti-phasic between the ears) after 2 s—the maximum possible IPD for a sinusoidal signal. This IPD transition—which was also applied at a minimum in the diotic AM cycle—elicited significant P1-N1-P2 responses for carrier frequencies between 500 to 1000 Hz. However, it is not clear whether the elicited response reflects binaural processing, a non-linear central summation of the monaural inputs from each ear, or a combination of both. Moreover, even if the response reflects true binaural processing, a transition from zero to anti-phasic IPD may actually reflect the binaural activity of neurons dedicated to processing interaural level differences (ILDs) i.e. likely LSO neurons. Activation of ipsilateral and contralateral LSO is similar when no ILD is present but increases in the ipsilateral LSO (relative to the ear in which the sound is more intense) and decreases in the contralateral LSO, as an ILD is applied (Boudreau 1967; Caird and Klinke 1983; Sanes 1990; Park et al. 1996; Tollin and Yin 2002; Tollin and Yin 2005). For low-frequency tones with IPD, instantaneous fluctuations in ILD alternate around zero ILD and the magnitude of these fluctuations are proportional to the IPD magnitude showing a maximum at 180 °. To this end, the magnitude of ILD fluctuations alternates synchronously between zero (0 ° IPD) and its maximum (180 ° IPD). LSO neurons could follow the regular change in ILD fluctuations, and this might contribute to the evoked response observed by Ross et al. (2007b).

In contrast, a benefit of our IPM stimulus is that the symmetrical IPD transitions produce ILD fluctuations that are invariant throughout the entire stimulus so that the observed IPM-FR should primarily reflect the response of neurons dedicated to processing ITDs in the temporal fine-structure of sounds—that is, likely the activity of MSO neurons (Goldberg and Brown 1969; Yin and Chan 1990; Spitzer and Semple 1995; Grothe and Park 1998; Brand et al. 2002).

### Fine Structure and Envelope ITD

Our stimuli contain two sources of contradictory ITD information—the dichotic carrier ITD and the diotic envelope (with no ITD). Carrier and envelope cues presented in opposing directions may “trade off” to create a centralized percept. Such “trading” is attributed to MSO neurons encoding carrier “fine structure”, and LSO neurons encoding the envelope (Joris and Yin 1995; Joris 1996). Whilst the lateralization and/or the salience of IPMs in the current stimuli could be influenced by the integration of these two conflicting ITD cues, importantly, the symmetrical phase reversal at each IPM and the diotic envelope means that neither instantaneous ILD cues nor envelope IPD cues were available to the listener. Indeed, Dietz et al. (2009) showed that a diotic envelope (i.e. one with no IPD) has effectively no influence on lateralization judgments based on carrier IPD. Similarly, in a study using the mismatch negativity (MMN) evoked potential, Schroger (1996) found that the response elicited by a combined change in ITD and ILD was comparable to the sum of the responses elicited by each change individually (i.e. ITD + ILD), suggesting that the two binaural cues are processed independently. From these studies, it seems right to expect that such trading is effective only for a dichotic envelope.

### IPD Tuning and Hemispheric Asymmetry

Dietz et al. (2009) observed that a carrier IPD of 45.0 ° required the largest opposing envelope IPD for centralizing the intracranial image. They attributed this to a large population of neurons with maximal response to 45.0 ° IPD as reported by McAlpine et al. (2001) for neural recordings from the inferior colliculus (IC) of guinea pigs. Dietz et al. also observed that a carrier IPD of 90.0 ° required a large opposing envelope IPD to centre the sound image (see also Buell and Hafter (1991)). This is consistent with the observed effect of IPM depth on the magnitude of the IPM-FR in the current study (see Fig. 6). The largest IPM-FRs were generated for IPM depths between ±45.0 ° and ±112.5 °—response magnitude diminished at larger or smaller IPM depths. In line with evidence that the distribution of best IPDs of IC and MSO neurons is maximal for IPDs between 45.0 ° and 90.0 °, the magnitude of the largest IPM-FR may reflect the transition of activations between neurons responding maximally in each hemisphere.

Additionally, hemispheric differences in IPM-FRs (see Fig. 7) support the hypothesis that location is represented asymmetrically between hemispheres, with the right hemisphere being more selective to spatial information than the left (Salminen et al. 2009, 2010, 2015; Palom et al. 2005; Tiitinen et al. 2006; McAlpine 2005; Magezi and Krumbholz 2010). Similarly, ASSRs hemispheric differences are consistent with previous studies indicating that the ASSR is right-hemisphere dominant (Ross et al. 2005; Hine and Debener 2007; Poelmans et al. 2012).

### Sensitivity to ITD within the ethological range

Previous electrophysiology or brain-imaging studies of ITD sensitivity have provided objective markers of ITD sensitivity for stimuli in which binaural transitions are beyond the ethological range and the generated intracranial percept is diffuse. For example, the zero to 180 ° IPD transitions employed by Ross et al. (2007b) generate ITDs well beyond the ethological range for frequencies lower than ≈750 Hz (Feddersen 1957; Kuhn 1977; Middlebrooks 1999). Even for tone frequencies at which this is not the case (>≈750 Hz), the intracranial percept generated by interaurally anti-phasic tones can be highly variable, being reported as diffuse or as originating from both ears simultaneously (Blauert and Lindemann 1986; Hall et al. 2005).

Similarly, Dajani and Picton (2006) presented listeners with wideband noise containing periodic abrupt modulations between interaurally correlated and de-correlated noise. By analysing the temporal and spectral waveforms of evoked responses to interaural noise modulation rates in the range 2 to 128 Hz, they observed robust steady-state responses for noise modulation rates between 6 and 8 Hz, also confirmed by our initial report (McAlpine et al. 2016). Abrupt changes in the lateralization (left and right percepts) of an interaurally coherent noise—created by ±1 ms ITD transitions (beyond the ethological range)—elicited a similar following response. Although responses were clearly obtained by Dajani and Picton (2006), interaural delayed coherent noise generates a diffuse or split sound image, and its percept is entirely insensitive to changes in fine-structure ITD.

In conclusion, our study is the first to demonstrate a systematic sensitivity to IPMs which is reflected behaviourally. The majority of applied IPD magnitudes were restricted to the ethological range and for which most would evoke compact sounds images clearly lateralized to one side or the other.

Further, with IPM-FRs evident even for extremely small IPDs (equivalent to ±120.0 μs for the ±22.5 ° condition), our study suggests that threshold performance (roughly a factor of 2 lower than the smallest IPM depth we employed) might be detectable in the EEG signal.

### Applications in diagnostics and hearing technologies

The IPM-FR provides a robust measure of ITD processing in normal-hearing listeners and might become useful to study basic properties of the binaural system in normal-hearing listeners. But, IPM-FR might also find a clinical application if found to be reduced in listeners with poor speech understanding despite having normal pure-tone audiograms—“hidden hearing loss” (Furman et al. 2013; Schaette and McAlpine 2011; Bharadwaj et al. 2015). Behavioural studies suggest that IPD thresholds increase with age despite subjects having normal hearing (Hopkins and Moore 2011; King et al. 2014) and also correlate with speech in noise performance. Thus, reduced IPM-FRs could provide an early indicator of temporal deficits in binaural processing and temporal fine-structure processing. As suggested by several authors, IPD discrimination can be used as a measurement of temporal coding (Lacher-Fougère and Demany 2005; Strelcyk and Dau 2009; Hopkins and Moore 2011; King et al. 2014). This is because the neural coding of these cues relies on the precise synchronization of neural activity with the stimulus. Therefore, IPM-FRs may also act as an objective measurement of temporal fine structure, and a reduced IPM-FR amplitude might indicate a hearing problem, before elevated thresholds in the audiogram.

Finally, the IPM-FR could also be adapted to aid across-ear electrode matching in bilateral cochlear implant (CI) users; this is particularly important when fitting children or users unable to perform behavioural tasks.

CI users typically show poor ITD sensitivity, which may in part be due to an across ear positional mismatch, resulting in electrodes conveying the same frequency information to mismatched auditory nerve fibres (Smith and Delgutte 2007; He et al. 2012; Hu and Dietz 2015). As such, the IPM-FR is only expected when intercochlear stimulation sites are matched and could therefore potentially be used for interaural electrode matching—or even the extent to which processing on otherwise—matched electrodes might be improved for binaural benefit by quantifying and correlating changes in the IPM-FR with behavioural measures of binaural processing over time (ITD sensitivity as well as speech understanding in reverberant noise).

## Footnotes

1 This was likely due to a faulty connection (poor contact or electrolyte bridges) between the common mode sense (CMS) active electrode and the driven right leg (DRL) passive electrode. These electrodes form a feedback loop, which drives the average potential of the subject as close as possible to the analogue-to-digital converter reference voltage in the analogue-to-digital box (AD-box). This results in a better common mode rejection ratio at 50 Hz when compared with using normal ground electrodes with the same impedance and enables any electrode to be used as the reference in the post-processing stage. Therefore, electrolyte bridges or unstable electrode contacts result in partial to complete loss of signal and increased noise levels.

2 Pilots diotic control recordings using all phase changes as in dichotic conditions were similar, as such, only one diotic control was considered for the entire study.
